# Unusual Presentation of Nephroblastoma in an Adolescent: A Case of Initial Misdiagnosis as a Renal Abscess

**DOI:** 10.7759/cureus.96687

**Published:** 2025-11-12

**Authors:** Mohamad Jaber, Walid Alameh

**Affiliations:** 1 Urology, Lebanese University Faculty of Medicine, Beirut, LBN; 2 Urology, Sahel General Hospital-University Medical Center (UMC), Beirut, LBN

**Keywords:** adolescent kidney tumor, kidney tumor, nephric abscess, nephroblastoma, wilms tumor

## Abstract

We present the case of a 15-year-old girl who initially had fever, flank pain, and imaging findings consistent with a renal abscess. She was treated with antibiotics and improved clinically, but follow-up imaging showed incomplete resolution. After one year, she developed a recurrence, and a large renal mass was identified. Radical nephrectomy was performed, and histopathology confirmed a nephroblastoma tumor. This unusual presentation demonstrates that recurrent or incompletely resolving renal collections may hide an underlying malignancy. Clinicians should maintain suspicion for neoplastic causes in such scenarios, even in age groups where infection is more likely.

## Introduction

Nephroblastoma, or Wilms tumor, is the predominant malignant renal neoplasm in pediatric patients, constituting approximately 95% of renal tumors in children and about 6% of all juvenile malignancies [1]. Histologically, it is a triphasic neoplasm composed of blastemal, epithelial, and stromal elements. This is associated with several disorders resulting from WT gene mutations, such as Beckwith-Wiedemann syndrome, Denys-Drash syndrome, WAGR (Wilms tumor, aniridia, genitourinary anomalies, and intellectual disability) syndrome, and hemihypertrophy. The typical presentation is a discernible abdominal mass, accompanied by abdominal pain, gross hematuria, and hypertension [2]. Wilms tumor is diagnosed based on clinical presentation and radiographic features, with biopsy rarely required for confirmation. Advances in chemotherapy have led to excellent outcomes, with a five-year overall survival rate exceeding 90% in children [1]. On the other hand, it is uncommon in adolescents and is associated with a worse prognosis and reduced survival rates compared to younger children [3,4].

## Case presentation

This case involves a previously healthy 14-year-old girl with no prior hospitalization history, who arrived with a five-day history of non-radiating left flank discomfort, which has intensified, accompanied by a high-grade fever. No lower urinary tract symptoms were seen. Physical examination revealed discomfort solely at the left costovertebral angle, with no evidence of pelvic pain or abdominal distension.

**Figure 1:**
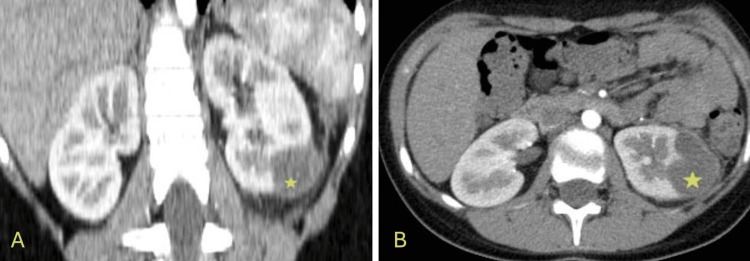
CT scan showing a 3.8cm lower pole left renal collection that is compatible with a renal abscess (yellow stars). A: Coronal view of the CT scan; B: Axial view of the CT scan

Laboratory results indicated a white blood cell count of 13,000 cells/µL and a C-reactive protein level of 9 mg/dl. Urinalysis revealed traces of hemoglobin, absent white blood cells, and a negative urine culture. The ultrasound with intravenous contrast revealed a 3.8 cm renal cortical fluid collection in the left lower pole, consistent with a renal abscess (Figure 1).

**Figure 2:**
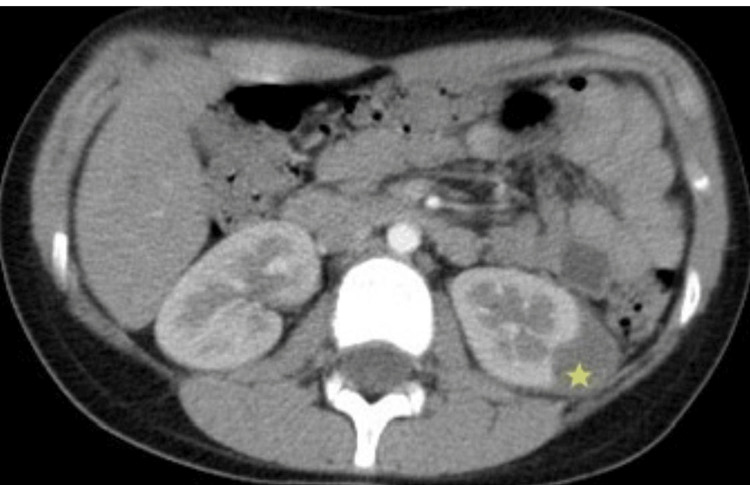
CT scan showing a reduction in size of the renal collection to 1.7 cm after two weeks of IV antibiotics (yellow star).

**Figure 3:**
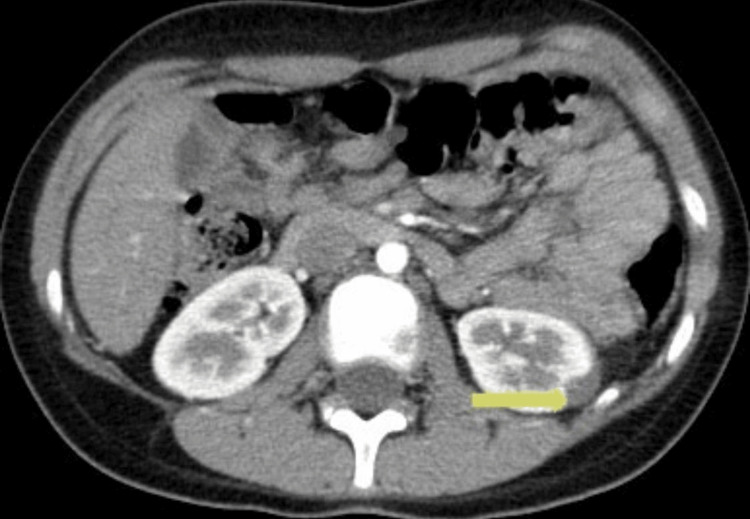
CT scan showing a reduction of the renal collection to 0.8 cm after three months of antibiotics treatment (yellow arrow).

The patient was initiated on intravenous meropenem and had clinical improvement after several days. The uroscan conducted after two weeks revealed a reduction in the extent of the renal fluid collection to 1.7 cm (Figure 2). Consequently, the patient was discharged with a prescription for intramuscular ertapenem for a further four weeks, followed by trimethoprim-sulfamethoxazole for two months. The uroscan was conducted again over the next two months, revealing a progressive reduction in the renal collection, which measured 0.8 cm after two months (Figure 3).

**Figure 4:**
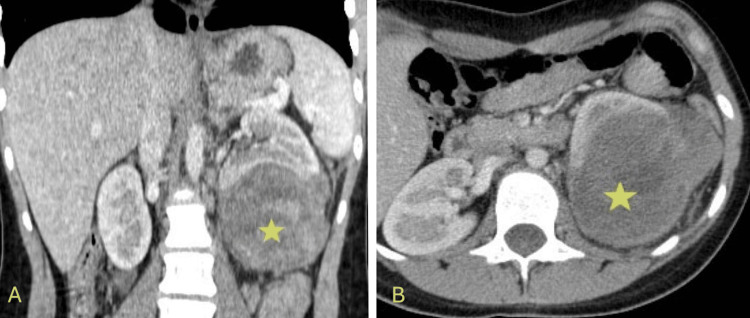
CT scan showing a left large renal mass that is occupying the mid and lower poles with heterogeneous enhancement (yellow stars). A: Coronal view of the CT scan; B: Axial view of the CT scan

One year later, the patient came with acute left flank discomfort, prompting the ordering of a uroscan with IV contrast, which revealed an 8 cm left kidney mass covering its mid and lower pole with heterogeneous enhancement (Figure 4). A choice for nephrectomy was made. During the procedure, the kidney exhibited fragility, accompanied by purulent exudate and necrosis. The specimen was dispatched to the pathology laboratory and revealed an undifferentiated renal tumor measuring 9 cm in greatest dimension. Immunohistochemical staining showed positivity for PAX8, vimentin, cytokeratin, glypican, and WT1, with focal positivity for CD57, while INI-1 was negative, which is compatible with nephroblastoma. The patient was kept in the hospital for five days postoperatively and discharged home without complication. The patient was referred to a pediatric oncologist and started on chemotherapy.

## Discussion

This case involves adolescent-onset nephroblastoma following a diagnosis and treatment for nephric abscess. Nephroblastoma is a solid malignant tumor of the kidney that originates from transformed primitive cells. It is classified as malignant due to its capacity for rapid growth and metastasis [5]. Wilms tumor is primarily a childhood malignancy, typically diagnosed before the age of four; however, a minority of cases may arise during adolescence [4].

This was not the first instance of this tumor exhibiting an atypical presentation. An 18-year-old female presented with a high-grade fever and right loin pain and was diagnosed with pyonephrosis, which required surgical exploration. Following a right subcapsular nephrectomy due to extensive renal damage, the diagnosis revealed a Wilms tumor of unfavorable histology [6]. A six-year-old girl with a history of recurrent pyelonephritis presented with left flank pain and fever. Imaging revealed acute pyelonephritis with a subcapsular collection consistent with a hematoma. Subsequent serial imaging indicated an increase in the collection and raised suspicion of a mass. A radical nephrectomy was performed, which confirmed the presence of intermediate-risk nephroblastoma [7]. A case involving a five-year-old girl presented with flank pain, fever, and a renal mass identified on CT scan as potentially a tumor or of infectious origin. A biopsy was performed, revealing no malignancy. Despite clinical improvement on antibiotics, the biopsy was repeated due to the persistence of the mass, ultimately leading to a diagnosis of Wilms tumor [8]. 

Following diagnosis post radical nephrectomy, the patient was referred to a pediatric oncologist to initiate adjuvant chemotherapy, in accordance with the National Comprehensive Cancer Network Guidelines (NCCN) guidelines, which recommend postoperative chemotherapy and radiotherapy in cases of high-grade or advanced-stage disease [9]. The initial presentation of high-grade fever accompanied by leukocytosis and a renal collection that does not enhance with IV contrast suggests a diagnosis of renal abscess, which measured less than 4 cm, thus negating the need for drainage; antibiotics alone were adequate for reduction. However, the persistence of a very small collection, measuring less than 1 cm, concealed a rapidly growing tumor that developed into a significant mass within less than one year.

Atypical presentations of nephroblastoma in adolescents pose diagnostic challenges due to their rarity in this age group, potentially leading to delays in diagnosis and treatment, thereby jeopardizing patient health through increased risk of metastasis. Consequently, given the exceptional rarity of renal abscess in otherwise healthy individuals under 18 years of age, a thorough assessment is necessary for any patient exhibiting symptoms of a kidney infection and a renal collection that does not completely resolve with antibiotic treatment, despite any observed partial improvement [10]. 

## Conclusions

Nephroblastoma is a malignant kidney tumor predominantly found in children, typically presenting as an abdominal mass. However, it can also manifest in adolescents and may be obscured by renal abscesses. Therefore, a high index of suspicion is essential for timely diagnosis and treatment.
